# Ammonium Inhibits Chromomethylase 3-Mediated Methylation of the *Arabidopsis* Nitrate Reductase Gene *NIA2*

**DOI:** 10.3389/fpls.2015.01161

**Published:** 2016-01-21

**Authors:** Joo Yong Kim, Ye Jin Kwon, Sung-Il Kim, Do Youn Kim, Jong Tae Song, Hak Soo Seo

**Affiliations:** ^1^Department of Plant Science and Research Institute for Agriculture and Life Sciences, Seoul National UniversitySeoul, South Korea; ^2^School of Applied Biosciences, Kyungpook National UniversityDaegu, South Korea; ^3^Plant Genomics and Breeding Institute, Seoul National UniversitySeoul, South Korea; ^4^Bio-MAX Institute, Seoul National UniversitySeoul, South Korea

**Keywords:** ammonium, CMT3, DNA methylation, gene expression, NIA2, nitrogen assimilation, plant growth

## Abstract

Gene methylation is an important mechanism regulating gene expression and genome stability. Our previous work showed that methylation of the nitrate reductase (NR) gene *NIA2* was dependent on chromomethylase 3 (CMT3). Here, we show that CMT3-mediated *NIA2* methylation is regulated by ammonium in *Arabidopsis thaliana*. CHG sequences (where H can be A, T, or C) were methylated in *NIA2* but not in *NIA1*, and ammonium [(NH_4_)_2_SO_4_] treatment completely blocked CHG methylation in *NIA2*. By contrast, ammonium had no effect on *CMT3* methylation, indicating that ammonium negatively regulates CMT3-mediated *NIA2* methylation without affecting *CMT3* methylation. Ammonium upregulated *NIA2* mRNA expression, which was consistent with the repression of *NIA2* methylation by ammonium. Ammonium treatment also reduced the overall genome methylation level of wild-type *Arabidopsis*. Moreover, CMT3 bound to specific promoter and intragenic regions of *NIA2*. These combined results indicate that ammonium inhibits CMT3-mediated methylation of *NIA2* and that of other target genes, and CMT3 selectively binds to target DNA sequences for methylation.

## Introduction

DNA methylation is an important epigenetic mechanism that regulates gene expression and genome stability in plants and animals (Herman and Baylin, 2003). DNA methylation in plants occurs at symmetric CG and CHG sequences and non-symmetric CHH sequences (where H can be A, C, or T). Three types of DNA methyltransferases (DNMTs) have been identified in plants, namely chromomethylase (CMT), methyltransferase (MET), and domain-rearranged methyltransferase (DRM) (Cao and Jacobsen, 2002b; Ronemus et al., 1996; Lindroth et al., 2001).

Chromomethylases contain six conserved motifs (I, IV, VI, VIII, IX, and X), a bromo adjacent homology (BAH) domain, and a chromodomain. The BAH domain is located at the N-terminal region, and the chromodomain is embedded between catalytic motifs I and IV. All DNMT1/MET1 proteins contain two BAH domains, whereas CMTs contain only one BAH domain. BAH domains are involved in protein–protein interactions, recognition of methylated histones, and nucleosome binding in animal systems (Armache et al., 2011; Kuo et al., 2012; Yang and Xu, 2013). Chromodomains function as methylated histone lysine-binding domains, facilitating recruitment to chromatin in animals (Sanchez and Zhou, 2009; Yap and Zhou, 2012). In plants, the BAH domain and chromodomain of CMT3 mediate its specific binding to histone H3 lysine 9 dimethylation (H3K9me2) (Du et al., 2012). Mutations in the BAH domain or chromodomain caused a failure of CMT3 binding to nucleosomes and a complete loss of CMT3 activity *in vivo*, suggesting that CMT3 associates with H3K9me2-containing nucleosomes through dual binding of its BAH and chromo domains to H3K9me2 to target DNA methylation.

*Arabidopsis* has three *CMT* genes, namely, *CMT1*, *CMT2*, and *CMT3* (Henikoff and Comai, 1998; Finnegan and Kovac, 2000; McCallum et al., 2000). In the ecotype Wassilewskija (WS), *CMT2* and *CMT3* are predicted to encode functional proteins (Henikoff and Comai, 1998), whereas *CMT1* encodes a non-functional protein (Jones et al., 2001). *CMT* genes have been identified in several plant species, including tobacco and maize, but they have not been identified in fungal or animal systems (Rose et al., 1998; Finnegan and Kovac, 2000). This indicates that CMT is a plant-specific DNMT. CMT2 and CMT3 have DNA methylation activity at CHG sites (Jones et al., 2001; Lindroth et al., 2001), and maintain CHH methylation at certain genomic loci (Cao and Jacobsen, 2002a; Cokus et al., 2008; Stroud et al., 2013). *Zea mays* methyltransferase 2 (ZMET2) and the *Nicotiana benthamiana* CMT3 homolog NbCMT3 were identified as CHG-specific DNMTs (Papa et al., 2001; Hou et al., 2013). MET1 is a homolog of mammalian DNMT1 that catalyzes symmetric CG methylation (Jones et al., 2001). MET1 has little effect on CHG methylation levels and it cannot substitute for CMT3 (Cokus et al., 2008; Stroud et al., 2013). The DRM DNMTs, which include DRM1, DRM2, and DRM3, have *de novo* DNA methylation activity at CG, CHG, and CHH sites through RNA-directed methylation pathways (Cao et al., 2003; Cokus et al., 2008; Henderson et al., 2010; Greenberg et al., 2011; Stroud et al., 2013). DRMs also maintain CHG and CHH methylation at specific genomic loci (Cao et al., 2003; Cokus et al., 2008; Henderson et al., 2010; Stroud et al., 2013). These combined results indicate that plant CHG and CHH methylation is controlled by CMT and DRM DNMTs (Chan et al., 2005; Stroud et al., 2013; Zemach et al., 2013).

The roles of plant MET1, DRM2, and CMT3 are well characterized. The expression patterns and levels of these DNMTs differ in different tissues and during different developmental stages. Mutation of these DNMTs causes severe loss of DNA methylation, which leads to abnormal development. For example, plants defective in CMT3 activity display abnormal embryo development (Pillot et al., 2010). Loss of MET1 activity delays flowering and reduces fertility, and these effects become more severe in the presence of CMT3 or DRM2 mutations (Xiao et al., 2006; Zhang and Jacobsen, 2006). A recent GUS study reported that these DNMTs are expressed in specific tissues or ubiquitously expressed at specific developmental stages (Huang et al., 2014). CMT3 is expressed only in specific organ regions that co-express DRM2 and MET1.

Nitrogen assimilation is a fundamental biological process that is essential for plant growth and development. Plants utilize nitrate as a source of environmental nitrogen, and nitrate is a potent signal that regulates nitrogen and carbon metabolism, plant growth, and development (Crawford and Forde, 2002; Forde, 2002; Stitt et al., 2002; Foyer et al., 2003). Nitrate is actively transported into cells from the soil by a nitrate transporter and then sequentially reduced to ammonia, which enters amino acid metabolic pathways primarily via the action of glutamine synthetase. NR, a key enzyme of the plant nitrogen assimilation pathway, forms homodimers that catalyze the NAD(P)H-dependent reduction of nitrate to nitrite (Campbell and Kinghorn, 1990; Solomonson and Barber, 1990). Therefore, the regulation of NR expression and NR activity is important for nitrate assimilation. A recent study reported that the *Arabidopsis* NRs NIA1 and NIA2 are positively regulated by sumoylation through the activity of the E3 SUMO ligase AtSIZ1 (Park et al., 2011). CMT3 sumoylation by AtSIZ1 is suggested to control NR gene expression (Kim et al., 2015a).

In the present study, we investigated the effect of ammonium on CMT3-mediated methylation of NR genes. We report that ammonium inhibits CMT3-mediated *NIA2* methylation without affecting on *CMT3* methylation. In addition, CMT3 binds to specific regions of *NIA2*. Our results provide evidence that CMT3-mediated *NIA2* methylation is negatively modulated by ammonium.

## Materials and Methods

### Plant Materials, Growth Conditions, and Ammonium Treatment

*Arabidopsis thaliana* Columbia-0 ecotype (wild-type, Col-0) was used in this study. For *in vitro* culture in artificial media, seeds were surface-sterilized for 10 min using commercial bleach containing 5% sodium hypochlorite and 0.1% Triton X-100, rinsed five times in sterilized distilled water, and then stratified for 3 days at 4°C in the dark. Seeds were then sown on agar plates containing Murashige and Skoog medium (pH 5.7), 2% sucrose, and 0.8% agar. For plants grown in a non-agar substrate, seeds were sown and grown on sterile vermiculite. All plants were grown in a growth chamber at 22°C under a 16 h light/8 h dark cycle. To examine the effect of ammonium, three different ammonium sources [(NH_4_)_2_SO_4_, NH_4_Cl, and NH_4_NO_3_] were used with similar results. Thus, (NH_4_)_2_SO_4_ was used for all experiments.

### Immunoprecipitation of Methylated DNA

Wild-type plants were grown in vermiculite for 3 weeks, treated with 5 mM (NH_4_)_2_SO_4_, and grown for an additional 12 h at 22°C. Then, genomic DNA was isolated from individual plants and sonicated to produce random fragments of 200–600 bp. The fragmented DNA (4 mg) was used in a standard methylated DNA immunoprecipitation (IP) (MeDIP) assay as described previously (Pomraning et al., 2009). Methylated DNA was recovered as described previously (Kim et al., 2015a).

### Illumina Genome Analyzer Sequencing

To perform second-strand synthesis of MeDIP-enriched ssDNA fragments, approximately 200 ng of MeDIP-enriched ssDNA fragments and 500 ng of random primers were mixed in a final volume of 57.9 μl, incubated at 70°C for 10 min, and then cooled gradually for 40 min. Subsequent experiments were performed as described previously (Kim et al., 2015a). End-repair of DNA fragments, addition of an adenine residue to the 3′ fragment ends, adaptor ligation, and PCR amplification using Illumina paired-end primers were performed as described previously (Pomraning et al., 2009). The products were analyzed by agarose gel electrophoresis, and bands were excised to produce libraries with 250–350 bp insert sizes, which were quantified using Quant-iT PicoGreen dsDNA Reagent and Kit (Invitrogen). Flow cells were prepared with 8 pM DNA according to the manufacturer’s recommended protocol and sequenced for 36 cycles on an Illumina Genome Analyzer II (Illumina). The obtained images were analyzed and base-called using GA pipeline software (version 1.3) with default settings (Illumina).

### Mapping Reads

The reads obtained from Illumina sequencing were mapped onto the *Arabidopsis* genome reference sequence (Bioconductor^1^) using Bowtie2 as described previously (Kim et al., 2015a). The sequence reads of untreated Col-0 wild-type plants were used as controls and to categorize the methylation sequences of (NH_4_)_2_SO_4_-treated wild-type plants.

### Bisulfite Sequencing

Genomic DNA was isolated from rosette leaves of 3-week-old wild-type plants treated with 5 mM (NH_4_)_2_SO_4_ or untreated plants (control). Bisulfite treatment and sample recovery were performed using the EpiTect Bisulfite Kit (QIAGEN) according to the manufacturer’s instructions. The primers were designed using MethPrimer software^2^. The percentage methylation (% C) was calculated as 100 × C/(C + T). Cytosine methylation in CG, CHG, and CHH contexts was analyzed and displayed using CyMATE (Hetzl et al., 2007).

### Analysis of CMT3 Pull-Down of *NIA2*

CMT3 binding to *NIA2* was examined by *in vivo* pull-down using a plant expression vector construct. The full-length *CMT3* cDNA was amplified by PCR using FLAG_4_-tagged forward and reverse primers, and inserted into the plant expression vector pBA002. This generated the construct *35S-CMT3-FLAG_4_*. Then, 3-week-old wild-type plants were infiltrated with transformed agrobacteria carrying *35S-CMT3-FLAG_4_* and sequentially treated with 5 mM (NH_4_)_2_SO_4_. Plants were incubated for 12 h at 22°C, and genomic DNA and total protein were extracted from rosette leaves of the transformed plants. CMT3-FLAG_4_ expression was examined by western blotting using an anti-FLAG antibody.

For the binding assay, genomic DNA was sonicated to produce random fragments of 200 - 600 bp. The fragmented DNA (4 mg) was used in the IP assay. Anti-FLAG antibody (20 μg) was added to each sample of fragmented DNA, reactions were brought to a final volume of 1 ml in IP buffer [10 mM sodium phosphate (pH 7.0), 140 mM NaCl, and 0.05% Triton X-100], and the mixture was incubated for 2 h at 4°C. Then, samples were incubated with 40 μl of protein A agarose beads for 12 h at 4°C and then washed seven times with 1 ml of IP buffer. DNA was recovered from the beads by phenol-chloroform extraction and ethanol precipitation. Purified DNA was amplified with specific primers for *NIA1* and *NIA2*, and then the DNA levels were examined by 1.2% agarose gel electrophoresis. The primers used for quantitative PCR were as follows: *NIA1*, 5′-GCTAGTAAGCATAAGGAGAG-3′ (forward) and 5′-CCTTCACGTTGTAACCCATCTTCT-3′ (reverse); *NIA2*, 5′-TGTCTCAGTACCTAGACTCTTTGC-3′ (forward) and 5′-TGTCTCAGTACCTAGACTCTTT-3′ (reverse).

The site of CMT3 binding to *NIA2* was determined using genomic DNA isolated from 3-week-old wild-type plants, which was sonicated to produce 200 - 600 bp fragments. The fragmented DNA (4 mg) was immunoprecipitated and analyzed as described above. Purified DNA was amplified with gene-specific primers, and the DNA levels were examined by 1.2% agarose gel electrophoresis. The following forward and reverse primers (21 oligonucleotides) were designed for PCR amplification of different regions (fragments) of *NIA2*: fragment - 3,000 to - 2,500 bp, 5′-TCAATAAGAGGAGGCCACAAA-3′ (forward) and 5′-ATTGTATTATATATATCAAAG-3′ (reverse); fragment - 2,499 to - 2,000 bp, 5′-CTGGCCAACATCTATTCATTA-3′ (forward) and 5′-ATATATATGATTTTTATATAC-3′ (reverse); fragment - 1,999 to - 1,500 bp, 5′-TGAAACTGCTATATGCAAGTA-3′ (forward) and 5′-CTAATTTTGGGTAACCAATAT-3′ (reverse); fragment - 1,499 to - 1,000 bp, 5′-AAGTTCACAAGAAAATCAATA-3′ (forward) and 5′-TTTCTTATTAAACGTTATTTT-3′ (reverse); fragment - 999 to - 500 bp, 5′-ATAAATATTGTATGATTATTA-3′ (forward) and 5′-TTTTCCTTTTTATTTTAGTCG-3′ (reverse); fragment - 499 to - 1 bp, 5′-TGTTTTGATCACATTTTTATA-3′ (forward) and 5′-TTGGAAAGTGTATAATCGTAA-3′ (reverse); fragment 1 to 500 bp, 5′-CATGGCGGCCTCTGTAGATAA-3′ (forward) and 5′-GTTTGACGAATCCGGTCACCT-3′ (reverse); fragment 501–1,000 bp, 5′-GGCCCATGAAATTCACCATGG-3′ (forward) and 5′-GTTGTCCTTGAAATGGTAGAA-3′ (reverse); fragment 1,001–1,500 bp, 5′-AGAGTTTTACCTTCTTTGGTA-3′ (forward) and 5′-ACCTCCACACGGGTCACTTTT-3′ (reverse); fragment 1,501–2,000 bp, 5′-CACGGTAGATGGTGGAGAGAC-3′ (forward) and 5′-ATCGTGTACAATCATAGATAT-3′ (reverse); fragment 2,001–2,500 bp, 5′-TCCTTATGGATCACCCGGGTG-3′ (forward) and 5′-TGGGTGGACACCGCCAAAGTA-3′ (reverse); fragment 2,501–3,412 bp, 5′-AGATTCCCTAACGGCGGGCTC-3′ (forward) and 5′-ATGAAAAAATGAACATATTCA-3′ (reverse).

### Examination of DNA Methylation by McrBC-PCR

The methylation analysis procedure involved 5-methylcytosine-specific restriction enzyme (McrBC)-based PCR using a previously published protocol (Lippman et al., 2003). Genomic DNA (500 ng) was isolated from wild-type plants treated with 5 mM (NH_4_)_2_SO_4_ or untreated plants (control) and digested with 30 units of McrBC endonuclease (New England Biolabs) for 3 h at 37°C. Then, quantitative PCR analyses were performed as described previously (Kim et al., 2015a). The primers used for quantitative PCR were as follows: *NIA1*, 5′-GCTAGTAAGCATAAGGAGAG-3′ (forward) and 5′-CCTTCACGTTGTAACCCATCTTCT-3′ (reverse); *NIA2*, 5′-TGTCTCAGTACCTAGACTCTTTGC-3′ (forward) and 5′-TGTCTCAGTACCTAGACTCTTT-3′ (reverse); *CMT3*, 5′-GCTAGTAAGCATAAGGAGAG-3′ (forward) and 5′-CCTTCACGTTGTAACCCATCTTCT-3′ (reverse); tubulin, 5′-GTGAGCGAACAGTTCACAGC-3′ (forward) and 5′-TTATTGCTCCTCCTGCACTT-3′ (reverse).

### Transcript Level Analysis for Genes Involved in NR Pathways and *CMT3*

Wild-type plants were grown for 3 weeks in vermiculite and treated with or without 5 mM (NH_4_)_2_SO_4_ for 12 h. Total RNA was extracted from rosette leaves, quantified, and divided into equal amounts. First-strand cDNA was synthesized from 5 μg of total RNA using an iScript cDNA Synthesis Kit (Bio-Rad), and cDNA was amplified by real-time qRT-PCR (MyiQ, Bio-Rad) according to the manufacturer’s instructions. PCR analysis and sequencing were performed as described previously (Kim et al., 2015b). The primers used for quantitative PCR were the same as those described in the previous section.

## Results

### Ammonium Reduces Genome Methylation Levels

Nitrogen is an essential element for plant growth, and plants take up nitrogen as nitrate or ammonium through roots or leaves to support biosynthetic production. Nitrate, the uptake of which is mediated by nitrate transporters, is converted to ammonia by NR and nitrite reductase, indicating that ammonia affects plant development as an end product before its incorporation into glutamine by glutamine synthase. Therefore, we first examined the effect of ammonium on genome methylation. For this experiment, wild-type plants were grown for 3 weeks in vermiculite and treated with or without 5 mM (NH_4_)_2_SO_4_ before being subjected to MeDIP sequencing analysis to determine the methylation level of the whole genome. The results showed that CpG and C methylation were lower in (NH_4_)_2_SO_4_-treated plants than in control plants (**Table 1**).

**Table 1:** CpG and C enrichment analysis of ammonium-treated plants by MeDIP sequencing.

	No treatment	(NH_4_)_2_SO_4_
CG regions	15,407,837	13,311,909
C regions	105,277,236	98,704,801
Total base(bp)	3,101,970,144	3,938,852,604
Alignment rate(%)	79.67	91.72

**FIGURE 1:**
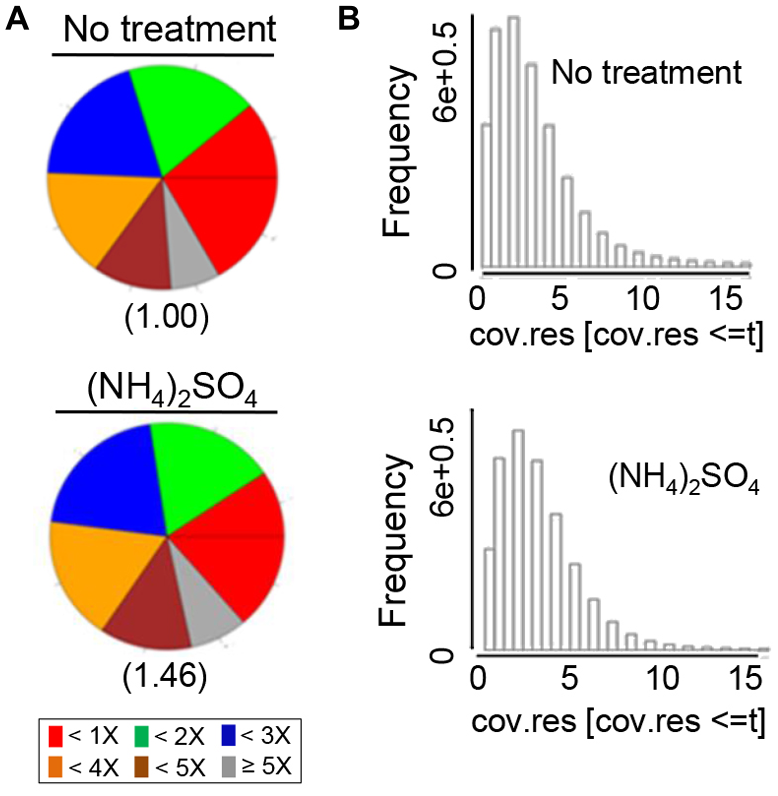
**MeDIP sequencing was used to detect whole genome methylation in ammonium-treated and untreated control *Arabidopsis* plants**. Genomic coverage was quantified by the number of DNA methylation measurements that overlapped with CpG islands. **(A)** CpG coverage was determined by counting the number of reads. CpG islands were identified using the program MEDIPS. The numbers below the diagrams indicate “pattern not covered.” **(B)** The methylation pattern is presented as sequence pattern coverage. The parameter *t* indicates the maximal coverage depth to be plotted (15 reads per sequence pattern).

**FIGURE 2:**
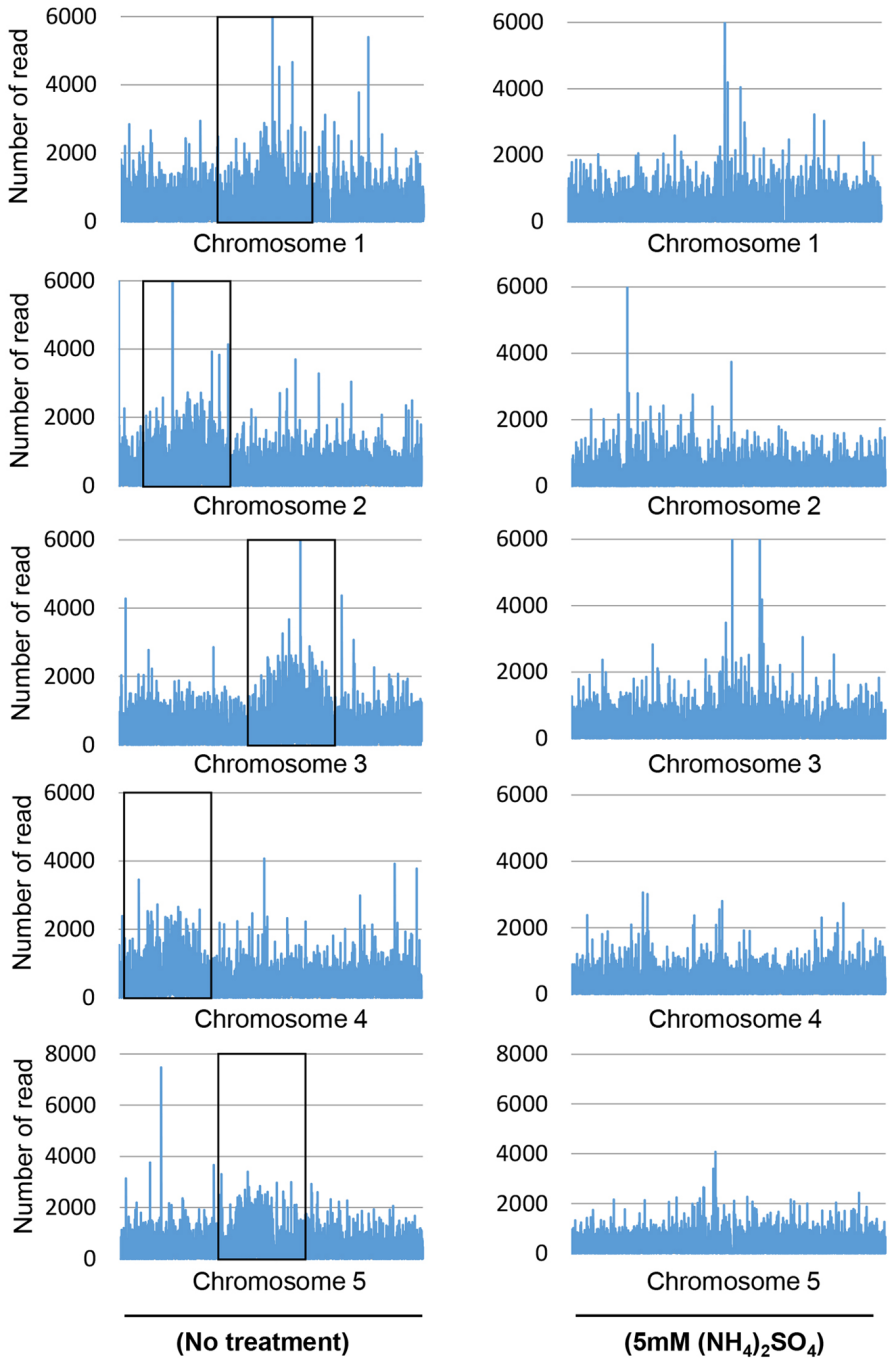
**DNA methylation landscape in the genome of ammonium-treated plants**. MeDIP sequencing was performed using whole genomic DNA isolated from wild-type and 5 mM (NH_4_)_2_SO_4_-treated plants. Each peak shows the distribution of sequenced read count in chromosome. Boxes indicate heterochmatic regions in each of the five chromosomes.

The number of reads that overlapped with CpG islands was determined to quantify the genomic coverage, which showed that the CpG coverage and sequence pattern coverage were lower in (NH_4_)_2_SO_4_-treated plants than in control plants (**Figure 1**). Therefore, the chromosomal distribution of methylated DNA was analyzed in wild-type plants treated with and without 5 mM (NH_4_)_2_SO_4_. Extensive DNA methylation was detected in the heterochromatic regions of each of the five chromosomes of control plants, although some noisy peaks were detected in other regions (**Figure 2**, left panel). This methylation pattern was similar to that reported previously (Zhang et al., 2006). In (NH_4_)_2_SO_4_-treated plants, methylation levels in the heterochromatic regions were lower than those in control plants (**Figure 2**, right panel). A comparison of whole genome methylation indicated that the methylation levels of 6,070 gene loci were downregulated in (NH_4_)_2_SO_4_-treated plants compared with those in control plants (Supplementary Table S1). This suggests that gene methylation status can be regulated by ammonium and lead to the upregulation of gene expression.

Similar results were obtained by performing replicate experiments using other ammonium sources including NH_4_Cl, indicating that changes in DNA methylation levels in response to treatment with 5 mM (NH_4_)_2_SO_4_ are caused by ammonium and not by sulfate.

### Ammonium Treatment Reduces *NIA2* Methylation Levels

MeDIP sequencing analyses indicated that the whole genome methylation level was lower in (NH_4_)_2_SO_4_-treated plants than in control plants. Therefore, we examined the methylation level of *NR* genes, which encode the first enzyme in the nitrate reduction pathway. The *Arabidopsis* genome database contains two NR genes, *NIA1* and *NIA2*, and their methylation levels were analyzed by bisulfite sequencing of genomic DNA isolated from control and (NH_4_)_2_SO_4_-treated plants. The results showed that *NIA1* and *NIA2*CG methylation levels were lower in (NH_4_)_2_SO_4_-treated plants than in control plants (**Table 2**; Supplementary Table S2). CHG methylation of *NIA1* was not detected in control and (NH_4_)_2_SO_4_-treated plants, whereas CHG methylation of *NIA2* was significantly reduced from 11.11 to 0% in response to (NH_4_)_2_SO_4_ treatment (**Table 2**; Supplementary Table S2). CHH methylation was slightly increased from 0 to 1.28% in *NIA1* in response to (NH_4_)_2_SO_4_, whereas CHH methylation was not detected in *NIA2* even after (NH_4_)_2_SO_4_ treatment (**Table 2**; Supplementary Table S2). The overall *NIA2* methylation level was reduced by (NH_4_)_2_SO_4_ treatment, but this had no effect on *NIA1* methylation (**Table 2**; Supplementary Table S2). The same results were obtained in replicate experiments with other ammonium sources.

**Table 2:** Methylation analysis of *NIA1* and *NIA2* genes by bisulfite sequencing after ammonium treatment.

**(A) *NIA1***			**(B) *NIA2***		
**No treatment**			**No treatment**		
	**Methylated (%)**	**Non-methylated (%)**		**Methylated (%)**	**Non-methylated (%)**
	
CG	10.00	90.00	CG	11.11	88.88
CHG	0.00	100.00	CHG	11.11	88.88
CHH	0.00	100.00	CHH	16.66	83.33
All	1.89	98.11	All	14.81	85.19
	
**(NH_**4**_)_**2**_SO_**4**_**			**(NH_**4**_)_**2**_SO_**4**_**		
	**Methylated (%)**	**Non-methylated (%)**		**Methylated (%)**	**Non-methylated (%)**
	
CG	5.55	94.44	CG	5.88	94.11
CHG	0.00	100.00	CHG	0.00	100.00
CHH	1.28	98.71	CHH	0.00	100.00
All	1.90	98.10	All	1.01	98.99

*The region of *NIA1* corresponds to positions from 1751st to 2128th (from transcription start site), and the region of *NIA2* corresponds to positions from 1552nd to 1898th (from transcription start site)*.

### Ammonium Inhibits CMT3 Binding to *NIA2*

**FIGURE 3:**
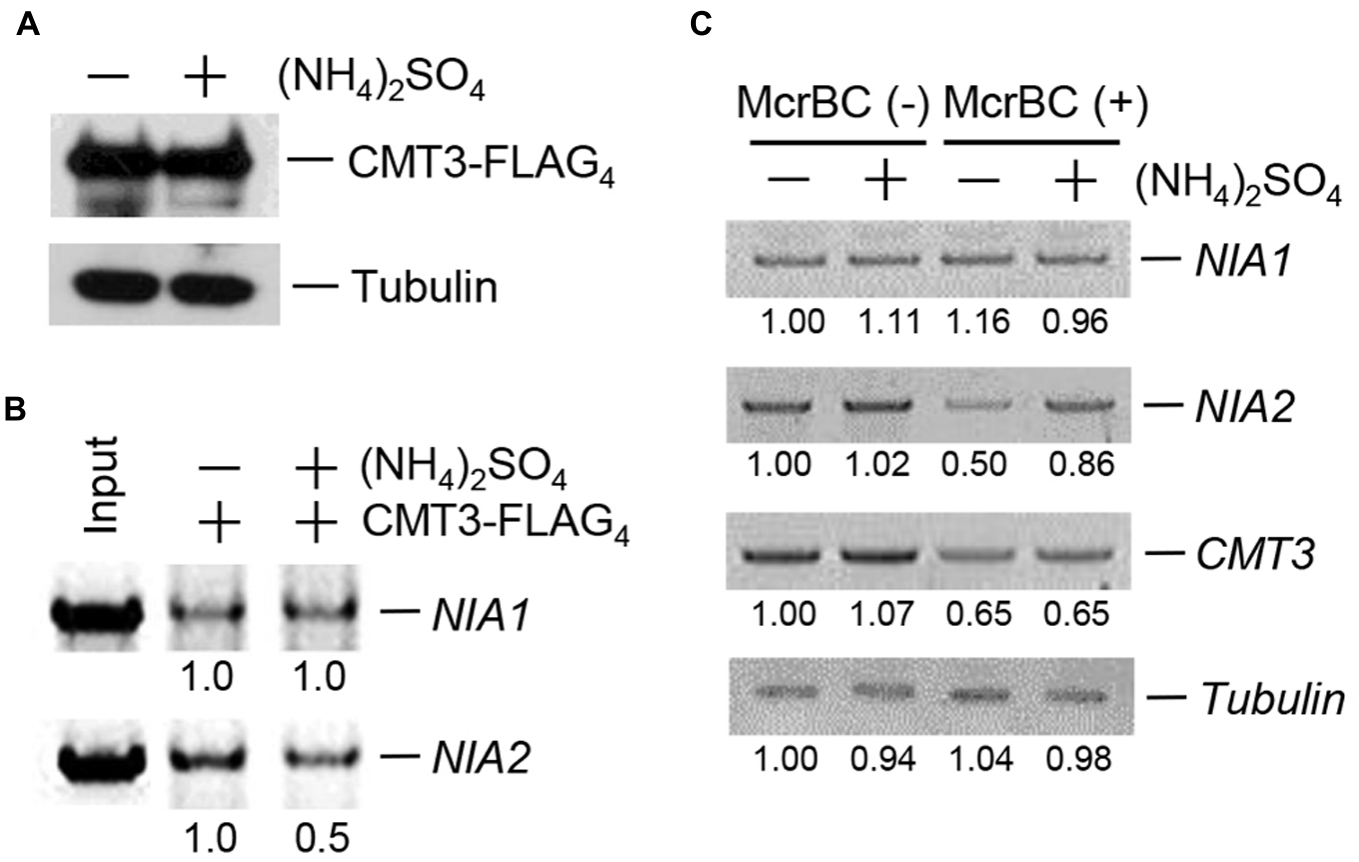
**CMT3 binding to *NIA2* decreased in response to ammonium**. **(A)** Wild-type *Arabidopsis* was infiltrated by *Agrobacteria* transformed with *35S-CMT3-FLAG_4_*, and then treated with or without 5 mM (NH_4_)_2_SO_4_. CMT3-FLAG_4_ expression was examined by western blotting with anti-FLAG antibody. Tubulin was used as the loading control. **(B)** CMT3-FLAG_4_ was pulled down with an anti-FLAG antibody, and DNA was extracted. The purified DNA was analyzed by PCR using gene-specific primers, and *NIA1* and *NIA2* levels were examined by agarose gel electrophoresis. Numbers under lanes indicate relative intensities. DNA levels were normalized to a value of 1.00 for DNA level in the “-” (NH_4_)_2_SO_4_ lane in each panel. **(C)** Genomic DNA was isolated from wild-type *Arabidopsis* treated with or without 5 mM (NH_4_)_2_SO_4_ and then digested with McrBC. The remaining DNA was used for PCR amplification with specific primers for *NIA1*, *NIA2*, and *CMT*3. Numbers under lanes indicate relative intensities. DNA levels were normalized to a value of 1.00 for DNA level in the “-” McrBC and “-” (NH_4_)_2_SO_4_ lane in each panel. Tubulin was used as the loading control.

The complete loss of CHG methylation in *NIA2* in response to (NH_4_)_2_SO_4_ led us to investigate the effect of (NH_4_)_2_SO_4_ on CMT3 binding to *NIA2*. The plant expression vector *35S-CMT3-FLAG4* was constructed and introduced into *Agrobacterium*, which was used to transform 3-week-old wild-type plants by infiltration. Then, CMT3-FLAG_4_ expression was analyzed in transformed plants after sequential treatment with 5 mM (NH_4_)_2_SO_4_ for 12 h and isolation of genomic DNA and total proteins. Western blot analysis showed that CMT3-FLAG_4_ was expressed in both untreated control and (NH_4_)_2_SO_4_-treated plants (**Figure 3A**). On the basis of this result, a DNA/protein-binding assay was performed using sonicated genomic DNA and an anti-FLAG antibody, and the pull-down DNA amount was estimated by PCR analysis with gene-specific primers for *NIA1* and *NIA2*. The results showed that complex formation between CMT3 and *NIA2* was inhibited in (NH_4_)_2_SO_4_-treated plants, whereas complex formation between CMT3 and *NIA1* was not affected by (NH_4_)_2_SO_4_ treatment (**Figure 3B**).

The methylation levels of *NIA1*, *NIA2*, and *CMT3* in (NH_4_)_2_SO_4_-treated plants were examined by McrBC digestion and PCR analysis with gene-specific primers. The gels showed similar band intensities for *NIA1* and *CMT3* in untreated control and (NH_4_)_2_SO_4_-treated plants, indicating that *NIA1* and *CMT3* methylation levels did not change in response to (NH_4_)_2_SO_4_ treatment (**Figure 3C**). However, the *NIA2* band intensity increased in (NH_4_)_2_SO_4_-treated plants, indicating that *NIA2* methylation was reduced in response to (NH_4_)_2_SO_4_ treatment (**Figure 3C**). Similar binding patterns and methylation levels were observed in response to treatment with other ammonium sources.

### Ammonium Upregulates *NIA2* Gene Expression

**FIGURE 4:**
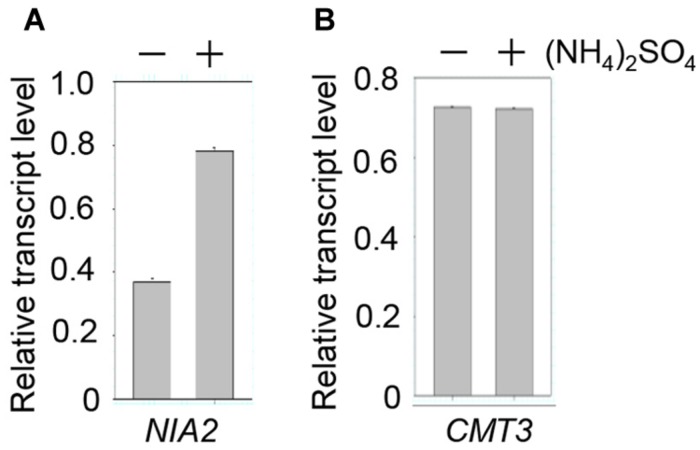
***NIA2* expression was upregulated by ammonium**. Total RNA was isolated from wild-type *Arabidopsis* plants treated with and without 5 mM (NH_4_)_2_SO_4_, and then subjected to real-time qRT-PCR analysis with specific primers for *NIA2* **(A)** and *CMT3* **(B)**.

Our results showed that *NIA2* methylation was inhibited by ammonium treatment, which suggests that ammonium induces *NIA2* expression. *NIA2* transcription was examined by qRT-PCR, which showed approximately 2-fold higher *NIA2* transcript levels in (NH_4_)_2_SO_4_-treated plants than in control plants (**Figure 4A**). This indicates that *NIA2* upregulation in (NH_4_)_2_SO_4_-treated plants resulted from low methylation levels caused by the lack of CHG methylation of *NIA2*. However, because low *NIA2* methylation levels could also be caused by low *CMT3* expression, we evaluated *CMT3* expression levels and detected no differences in *CMT3* transcript levels between (NH_4_)_2_SO_4_-treated and control plants (**Figure 4B**). These data indicate that low methylation levels and high transcription levels of *NIA2* in response to (NH_4_)_2_SO_4_ were caused by impaired CHG methylation of *NIA2*. Similar gene expression patterns were detected with other ammonium sources.

### CMT3 Binds to Specific Regions of *NIA2*

**FIGURE 5:**
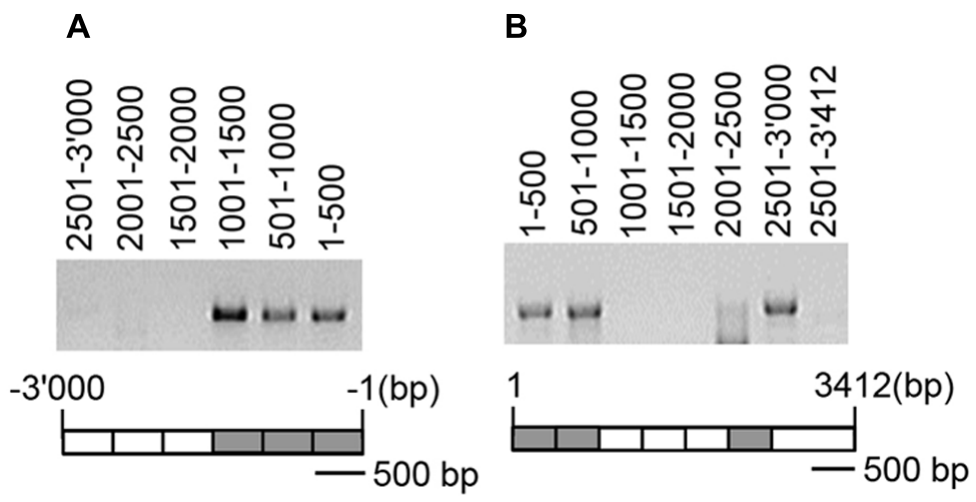
**CMT3 bound to specific regions of *NIA2***. Wild-type *Arabidopsis* was infiltrated with agrobacteria carrying *35S-CMT3-FLAG_4_*, and DNA/CMT3-FLAG_4_ complexes were pulled down with an anti-FLAG antibody. Genomic DNA was extracted from pulled-down samples and subjected to PCR analysis with gene-specific primers. **(A)** DNA fragments were PCR amplified with specific primers for the *NIA2* promoter, and the amplified DNA levels were examined by agarose gel electrophoresis. **(B)** DNA fragments were PCR amplified with specific primers for the *NIA2* coding region, and the amplified DNA levels were analyzed by agarose gel electrophoresis. The amplified DNA positions are illustrated under the agarose gels. Gray boxes indicate highly amplified DNA fragments; white boxes indicate very low levels of amplified DNA fragments or positions with no amplification.

CMT3 catalyzed the CHG methylation of *NIA2* but not that of *NIA1*. Therefore, the CMT3-binding region in *NIA2* was detected using IP and PCR analysis. Wild-type *Arabidopsis* plants were transformed by infiltration with *Agrobacteria* carrying *35S-CMT3-FLAG_4_*, and the DNA/CMT3-FLAG_4_ complex was pulled down with an anti-FLAG antibody. DNA was then amplified by PCR using specific primers for the promoter and coding regions of *NIA2*. The following three regions formed complexes with CMT3-FLAG_4_: the 1.5 kb promoter region from-1,500 to -1 bp (**Figure 5A**), the 1.0 kb coding region from 1 to 1,000 bp, and the 0.5 kb coding region from 2,501 to 3,000 bp (**Figure 5B**). These results indicate that CMT3 binds to specific regions in the *NIA2* promoter and coding sequence, suggesting that CMT3 catalyzes *NIA2* methylation at specific sequences or structures.

## Discussion

The current study aimed to define the role of CMT3 in nitrogen assimilation. We examined the effect of ammonium on whole genome methylation and observed that ammonium reduced the levels of whole genome methylation (**Table 1** and Supplementary Table S1). Plants utilize ammonium for carbon metabolism, nitrogen fixation, and biosynthesis of metabolic building blocks. Therefore, the effect of ammonium on reducing genome methylation suggests that high intracellular nitrogen concentration induces gene demethylation or represses gene methylation, thereby upregulating the expression of numerous genes involved in nitrogen metabolism. The carbohydrate-to-nitrogen (CN) ratio has a central and interactive role in regulating post-germination growth because the level of nitrogen is regulated with respect to that of carbohydrate during development (Huppe and Turpin, 1994). This suggests that ammonium treatment increases the intracellular nitrogen concentration, which affects nitrogen-sensitive metabolic networks. These combined data indicate that ammonium treatment induces the expression of genes involved in nitrogen assimilation by activating DNA demethylases or inactivating DNMTs.

CMT3-mediated genome methylation is a critical epigenetic modification that regulates plant growth and development. CMT3 functions in flower and reproductive organ development, embryogenesis, seed viability (Xiao et al., 2006; Pillot et al., 2010), organogenesis, and shoot regeneration from root explants (Shemer et al., 2015). CMT3 is involved in shoot growth regulation in *Arabidopsis* (Lin et al., 2015) and plays a role in tobacco leaf development by modulating the jasmonate pathway (Coustham et al., 2014). In addition, CMT3 acts as a functional regulator of oxidative stress to prevent genome instability and the accumulation of mutations caused by ionizing irradiation (Sidler et al., 2015). These data indicate that CMT3 catalyzes the methylation of numerous genes and may methylate and silence transposable elements (Kato et al., 2003). Therefore, CMT3 functions as a plant-specific DNMT that modulates growth, development, and differentiation throughout the plant life cycle from germination to seed maturation.

Our previous study showed that CHG methylation occurs on *NIA2* but not on *NIA1* and that CMT3 catalyzes CHG methylation of *NIA2* (Kim et al., 2015a). In the present study, CHG methylation of *NIA2* was not detected in ammonium-treated plants (**Table 2**), suggesting that ammonium treatment inactivates CMT3 activity or represses *CMT3* expression. The mechanism of CMT3 methylation of target genes has not been completely elucidated. Three possible mechanisms are proposed to explain how CMT3 distinguishes target DNA sequences. First, CMT3 may distinguish target genes through specific siRNAs in a siRNA/CMT3 complex, leading to CHG methylation of the specific target genes. This is similar to the recently reported DRM2 methylation of genes with target sequence homology to specific siRNAs as an AGO4/siRNA/DRM2 complex (Matzke and Mosher, 2014; Zhong et al., 2014). Since AGO4/siRNA complex formation is a prerequisite for the interaction of siRNAs with other components including methylases, identification of AGO4/siRNA complex interacting with CMT3 may provide the mechanism that CMT3 distinguishes target DNA sequences.

Second, the BAH domain and chromodomain of CMT3 recognize H3K9me2, leading to CMT3 binding to nucleosomes to methylate target DNA sequences (Du et al., 2012). This indicates that the BAH domain and chromodomain guide CMT3 to specific genomic regions by interacting with specific chromatin modifications. Therefore, examination of H3K9me2 of *NIA1* and *NIA2* genes *in vivo* may also provide clues as to why CMT3 selectively methylates the *NIA2* gene.

Third, the citrullination of DNMT3A by peptidylarginine deiminase (PADI4) stimulates its DNMT activity (Deplus et al., 2014). Citrullination affects protein structure and function (Lee et al., 2005; Gyorgy et al., 2006; Guo and Fast, 2011). CMT3 can be citrullinated by a plant PADI4 homolog. Therefore, CMT3 citrullination can modulate its ability to interact with partners involved in CMT3 targeting to specific genes and DNA methylation, suggesting that citrullination can help CMT3 discriminate among potential target sequences.

CHG methylation of *NIA2* in control wild-type plants but not in ammonium-treated plants (**Table 2**) indicated that CMT3-mediated CHG methylation of *NIA2* was blocked by ammonium treatment, suggesting that ammonium treatment represses *CMT3* expression. However, *CMT3* transcript levels and methylation levels were not affected by ammonium treatment (**Figure 4B**). This indicates that repression of CMT3-mediated CHG methylation of *NIA2* in response to ammonium treatment may not result from changes in CMT3 levels, but may be caused by changes in CMT3 activity due to post-translational modification. Our previous study showed that the DNMT activity of CMT3 was increased by sumoylation by the E3 SUMO ligase of AtSIZ1 (Kim et al., 2015a). This suggests that ammonium treatment may block AtSIZ1-mediated CMT3 sumoylation or other CMT3 modifications that are required for CMT3 activation.

CHG methylation correlates with H3K9m2 (Bernatavichute et al., 2008; Deleris et al., 2012), and the H3K9m2 histone MET triple mutant *kyp*/*suvh5*/*suvh6* shows a reduction in CHG methylation similar to that found in the *cmt3* mutant (Ebbs and Bender, 2006; Stroud et al., 2013). Some CHG sites also lost methylation in the *met1* mutant, indicating that CHG methylation partly depends on CG methylation (Stroud et al., 2013). A lack of CHG methylation correlates with a lack of the H3K9m2 marker, which is maintained by the histone methylase INCREASE IN BONSAI METHYLATION1 (IBM1) (Saze and Kakutani, 2011). These results suggest that the reduction in genome methylation levels in response to ammonium treatment can be affected by other DNA and histone METs. Therefore, further investigation of these mutants is needed to discover how ammonium regulates gene methylation or demethylation, including that of *NIA2*.

## Conclusion

The present study showed that ammonium has no effect on *CMT3* methylation and expression, whereas it blocks CMT3-mediated CHG methylation of *NIA2*, which induces *NIA2* expression. Future studies should be aimed at identifying CMT3-interacting factors, including siRNAs and proteins, and at examining the modulation of CMT3 DNMT activity by phytohormones or chemicals including ammonium. This may help elucidate specific mechanisms regulating CMT3-mediated genome methylation during specific growth and developmental stages, as well as during nitrogen assimilation.

## Author Contributions

HSS designed the studies. JYK, and DYK performed experiments. JYK, SIK, YJK, JTS, and HSS interpreted data. JYK and HSS wrote the manuscript. All authors commented on the results and the manuscript.

## Conflict of Interest Statement

The authors declare that the research was conducted in the absence of any commercial or financial relationships that could be construed as a potential conflict of interest.

## Footnotes

1 https://bioconductor.org/packages/release/data/annotation/html/BSgenome.Athaliana.TAIR.TAIR9.html

2 http://www.urogene.org/methprimer/
