# Supplementary material for: Ammonium Inhibits Chromomethylase 3-Mediated Methylation of the Arabidopsis Nitrate Reductase Gene NIA2
Source: Front Plant Sci. 2016 Jan 21;6:1161. doi: 10.3389/fpls.2015.01161 (PMC4720742; doi:10.3389/fpls.2015.01161)
Supplement: Supplementary file 3 [file Data_Sheet_1.DOCX]

**SUPPLEMENTARY MATERIAL**

**Title: Ammonium Inhibits Chromomethylase 3-Mediated Methylation of the *Arabidopsis* Nitrate Reductase Gene *NIA2***

Joo Yong Kim, Do Yeon Kim, Ye Jin Kwon, Sung-Il Kim, Jong Tae Song, Hak Soo Seo*****

*** Correspondence:** Hak Soo Seo, seohs@snu.ac.kr

| **Supplementary Table 1. Gene loci where the DNA methylation level was down-regulated in 5 mM (NH_4_)_2_SO_4_-treated plants.**  **Supplementary Table 2. Overall CG, CHG and CHH methylation sites of *NIA1* and *NIA2* genes in non-treated wild-type or 5 mM (NH_4_)_2_SO_4_-treated plants.** |  |
| --- | --- |
